# Octopus arms exhibit exceptional flexibility

**DOI:** 10.1038/s41598-020-77873-7

**Published:** 2020-11-30

**Authors:** E. B. Lane Kennedy, Kendra C. Buresch, Preethi Boinapally, Roger T. Hanlon

**Affiliations:** 1 Marine Biological Laboratory, 7 MBL St, Woods Hole, MA 02543 USA; 2 Northeastern University, 360 Huntington Ave, Boston, MA 02115 USA

**Keywords:** Biomechanics, Zoology, Behavioural methods

## Abstract

The octopus arm is often referred to as one of the most flexible limbs in nature, yet this assumption requires detailed inspection given that this has not been measured comprehensively for all portions of each arm. We investigated the diversity of arm deformations in *Octopus bimaculoides* with a frame-by-frame observational analysis of laboratory video footage in which animals were challenged with different tasks. Diverse movements in these hydrostatic arms are produced by some combination of four basic deformations: bending (orally, aborally; inward, outward), torsion (clockwise, counter-clockwise), elongation, and shortening. More than 16,500 arm deformations were observed in 120 min of video. Results showed that all eight arms were capable of all four types of deformation along their lengths and in all directions. Arms function primarily to bring the sucker-lined oral surface in contact with target surfaces. Bending was the most common deformation observed, although the proximal third of the arms performed relatively less bending and more shortening and elongation as compared with other arm regions. These findings demonstrate the exceptional flexibility of the octopus arm and provide a basis for investigating motor control of the entire arm, which may aid the future development of soft robotics.

## Introduction

Octopuses are soft-bodied molluscs with eight arms, each of which is lined with suckers along the oral surface. Each arm is thicker at its proximal base and tapers uniformly towards its distal tip^1^. Although octopuses have highly developed visual systems, they are largely tactile creatures^2^. The arms and suckers constitute most of the body mass and account for most of the neurons and muscles; the vast majority of their behaviours depend upon these appendages^3–6^. The arms and suckers are multi-functional; collectively they are used for locomotion, grasping, pushing, pulling, wrapping, twisting, and chemo-tactile sensing. Behaviourally, the arms are used for crawling, walking, disruptive camouflage, signalling, mimicry, capturing or extracting prey, fighting, and mating^2,7,8^.

Octopus arms and suckers are muscular hydrostats^1,9,10^. That is, they do not possess rigid structures, but instead rely on the control of internal pressure to create support and movement^11^. A constant volume is maintained because of densely arranged incompressible muscle tissues. Muscle control in three axes (parallel, perpendicular, and helical/oblique) and the lack of physical constraints from rigid components provide a theoretically unlimited range of movement along the entire length of such a structure. As noted by Yekutieli et al.^12^, “MH [muscular hydrostatic] skeletons have the greatest potential for localized, complex, and diverse movements—much greater than in other types of skeletons.” Examples of muscular hydrostats include vertebrate tongues and elephant trunks in addition to the arms and tentacles of cephalopods^13^.

**Figure 1:**
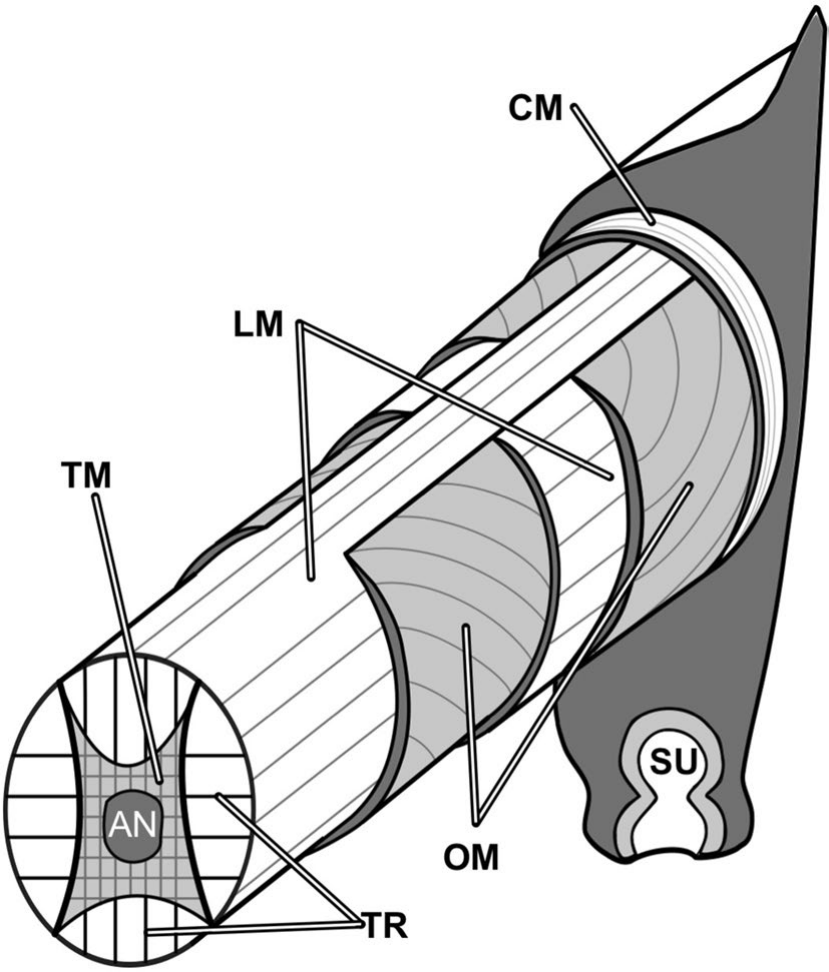
Simplified schematic diagram of the arm of an octopus showing the general three-dimensional arrangement of muscle fibres. A central mass of transverse muscle (TM) surrounds the axial nerve cord (AN) and extends peripherally in sheets called trabeculae (TR) to interdigitate with bundles of longitudinal muscle fibres (LM). Oblique muscle layers (OM), of both left and right handedness, are located on the lateral peripheries of the arm, separated by additional longitudinal muscle. SU, sucker; CM, circumferential muscle layer; TR, trabeculae—sheets of transverse muscle fibres that project through oblique and longitudinal muscle (modified from^16^).

Octopus arms are supported and articulated largely through concomitant activation of intrinsic muscle groups organized longitudinally, obliquely, and transversely^11^ (Fig. 1; see Kier and Stella^1^ for detailed descriptions of octopus arm intrinsic musculature)**.** Elongation and shortening are achieved by antagonistic activation of transverse and longitudinal muscles, respectively. Stiffening is achieved by co-activation of transverse and longitudinal muscles. Coordinated use of these two muscle groups produces bending when contraction of longitudinal muscle on the inside radius of the bend is accompanied by transverse muscle that provides resistance to longitudinal compression. Torsion (sometimes called twisting) results from the contraction of helically wound oblique muscles and the associated connective tissue array; octopuses have both right-handed and left-handed helical muscle groups along the length of the arm that enable torsion in both directions.

Throughout the substantial literature on the octopus arm, there is a common and often-mentioned assumption that octopus arms are “highly flexible,” have “virtually unlimited degrees of freedom,” and “behave as unrestricted classic muscular hydrostats”^7,14,15^. We set out to examine this assumption by calculating how diverse the arm deformations are in *Octopus bimaculoides*, while keeping in mind the assumption that each arm is *likely* to perform all four deformations based on published literature on anecdotal behavioural observations and muscle morphology^6,10,17^. Yet does this hold for all four arm pairs in this species, and does it apply along the length of each arm? At least one paper^10^ found structural variants among arm muscle groups in *O. vulgaris* suggesting that this may impose some biomechanical constraints on some aspect of arm flexibility. Moreover, even if arm muscle morphology is the same along each arm, the neural control components may be wired differently so that highly diverse flexibility is not distributed equally on each part of each of the eight arms. While there are more than 300 species of octopus^18^ with an enormous diversity of body morphometrics, *O. bimaculoides* is typical of many of these species in terms of size and body proportions so it can provide a baseline from which to assess other species^1^. Our approach for this observational study was to videotape a wide range and large number of arm movements in *O. bimaculoides* and determine visually how flexible each arm is along its length by recording deformation types for each arm region for all eight arms. By “flexible” we are not referring to the range of movement but rather to the nature of mobility of any part of the arm. This method, which is simple and straightforward, allowed us to process a larger data set and address a basic question about arm flexibility that more sophisticated methods have not yet addressed.

## Methods

Ten wild-caught *O. bimaculoides* were used in this study: seven males and three females. They were collected off the coast of southern California and transported to the Marine Biological Laboratory in Woods Hole, MA. Animals were housed individually in glass aquaria enriched with clay-pot dens and artificial plants. Animals were fed live mussels, shrimp, and crabs. Animal sizes ranged from ca. 400—700 g.

**Figure 2:**
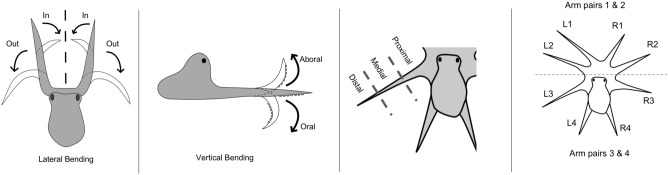
Observation methodology definitions. Diagrams illustrating bending directions, arm region categories, and arm numbering.

To elicit a wide variety of arm movements, several tanks were used to record video to enable observations of arm use in different scenarios: (1) tanks without any barriers to allow the octopus to move freely, (2) tanks with clear plexiglass barriers containing a single hole through which one or two arms could fit and (3) a tank with a clear plexiglass barrier containing many holes. Various objects (plastic jack, plastic clip, textured cylinder, rounded bulb, rubber stopper) or food items on the end of plastic rods were presented by hand to attract the attention of the octopus and elicit a behavioural response, resulting in a wide variety of arm movements. Arm movements were videotaped over many months using either one or two cameras from a variety of angles. Animals were given time to interact with the objects to broaden the potential for the arm to move in all directions with respect to the deformations being observed. For all eight arms, all completely visible occurrences of four deformation types were recorded: bending (orally, aborally, inward, outward), torsion (clockwise and counter-clockwise), elongation, and shortening (Fig. 2). Arm deformations created by incidental researcher manipulation of the presented objects were not recorded. Occurrences of elongation and shortening were determined visually by a noticeable difference in spacing between suckers in addition to the thickness of the arm segment. As muscular hydrostats, an increase in length resulted in a decrease in thickness, whereas shortening resulted in an increase in thickness. This visual method of analysis did not allow for precise measurement of bending angles, so the directions of bending deformations were recorded as the closest approximation to one of the four directions. The location of each deformation was recorded as being in the proximal, medial, or distal third of the arm (Fig. 2) and whether the suckers were in contact with another surface within that deformation. Sucker-independent deformations were defined as those that occurred in open space without the suckers touching anything. Although stiffening is recognized as a critical additional deformation^1,13^, it was not feasible to judge this characteristic visually from camera recordings. Footage was trimmed to remove periods of inactivity and visual obstruction before analysis. Three trained observers (not involved in the filming) recorded observational data into a single collaborative document after reviewing the collected footage together and comparing observations. Before data collection began, all observers reviewed multiple clips together to establish agreement in grading methods. During data collection, observers frequently met as a group to confirm grading methods and address any individual concerns. A subset of eight short clips was used to test for inter-observer reliability using Pearson correlation coefficients^19^ averaged with Fisher z’ transforms^20,21^; the average correlation was calculated to be *r* = 0.842, which indicates acceptable reliability^19^ of scoring among the three observers. To compare counts of deformations for arm pairs and regions, we used a Pearson Chi Square test with Bonferroni Adjustment; post hoc comparisons were made using adjusted residuals. Invertebrates are not included in laboratory animal welfare regulations in the USA; therefore an animal use protocol was not required for this study.

## Results

A total of 16,563 observations of arm deformations were recorded from 120 min of video footage. All four deformation types (bending, torsion, shortening, elongation) were observed in all eight arms and in all three arm regions (proximal, medial, distal).

### Bending

**Figure 3:**
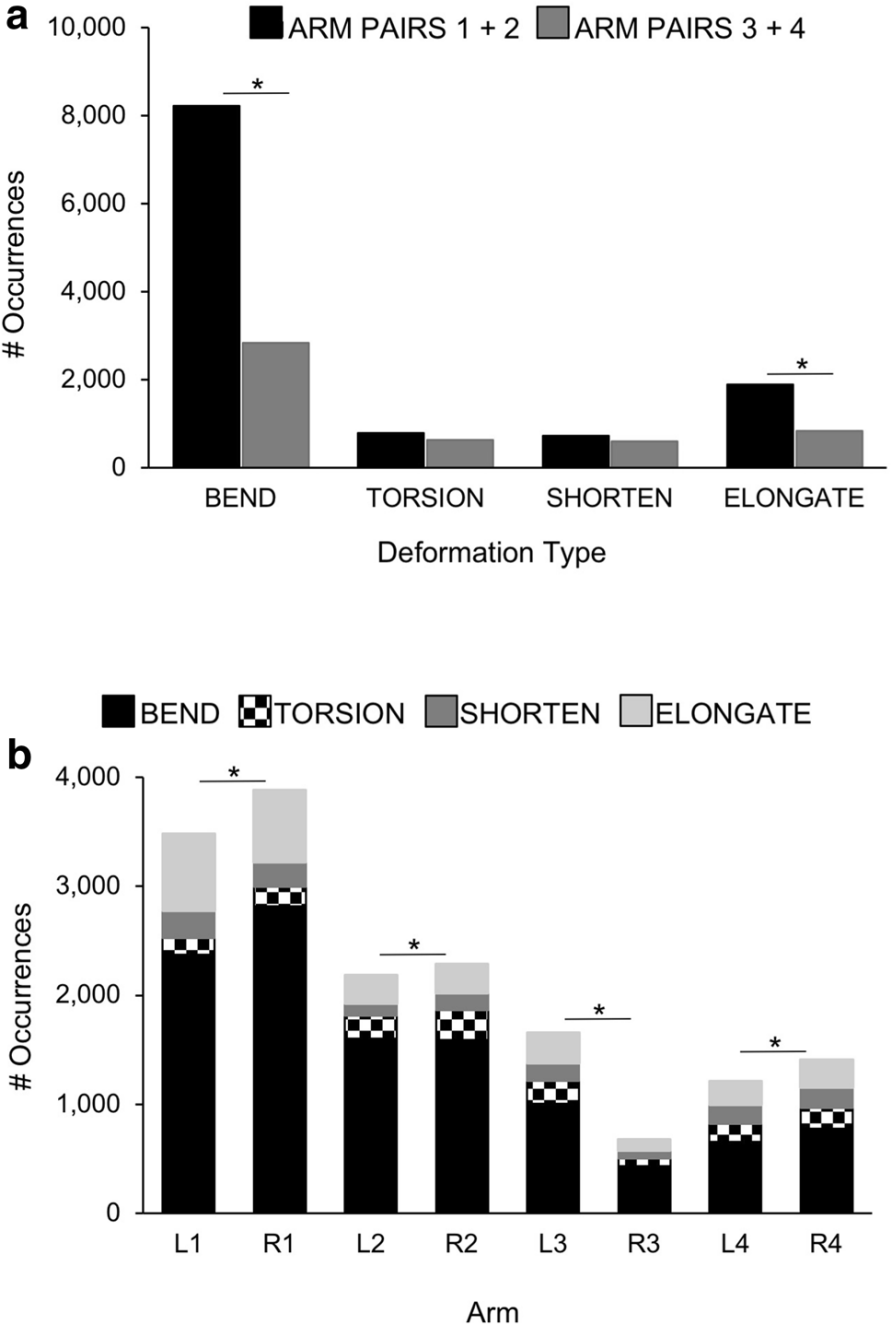
Distribution of deformations among arms of *O. bimaculoides*. **(A)** Comparisons of anterior and posterior arm deformations. The number of occurrences of each deformation type (bending, torsion, shortening, and elongation) are plotted with paired bars comparing anterior arm pairs (1 and 2) and posterior arm pairs (3 and 4). Bending was the most common arm deformation: more than 11,000 examples were observed, the vast majority occurring in the anterior arms (X^2^ = 252.56, p = 0.006). Elongation was observed 2,723 times and was seen more often in anterior than posterior arm pairs (X^2^ = 112.40, p = 0.003). Shortening was observed 1,340 times; no significant differences were found in anterior versus posterior arm pairs (X^2^ = 0.34, p = 0.55). Torsion was observed 1,426 times; no significant differences were found in anterior versus posterior arm pairs (X^2^ = 0.74, p = 0.39). **(B)** Distribution of deformations among all eight arms of *O. bimaculoides*. Stacked bars depicting the distribution of occurrences of all four deformation types (bending, torsion, elongation, and shortening) for each arm. All deformations occurred more frequently on the right than on the left side of the body (X^2^ = 10.49, p = 0.015), with the exception of the hectocotylized arm in males (R3; p = 0.001). Asterisks indicate significant differences in arm use at p < 0.01.

**Figure 4:**
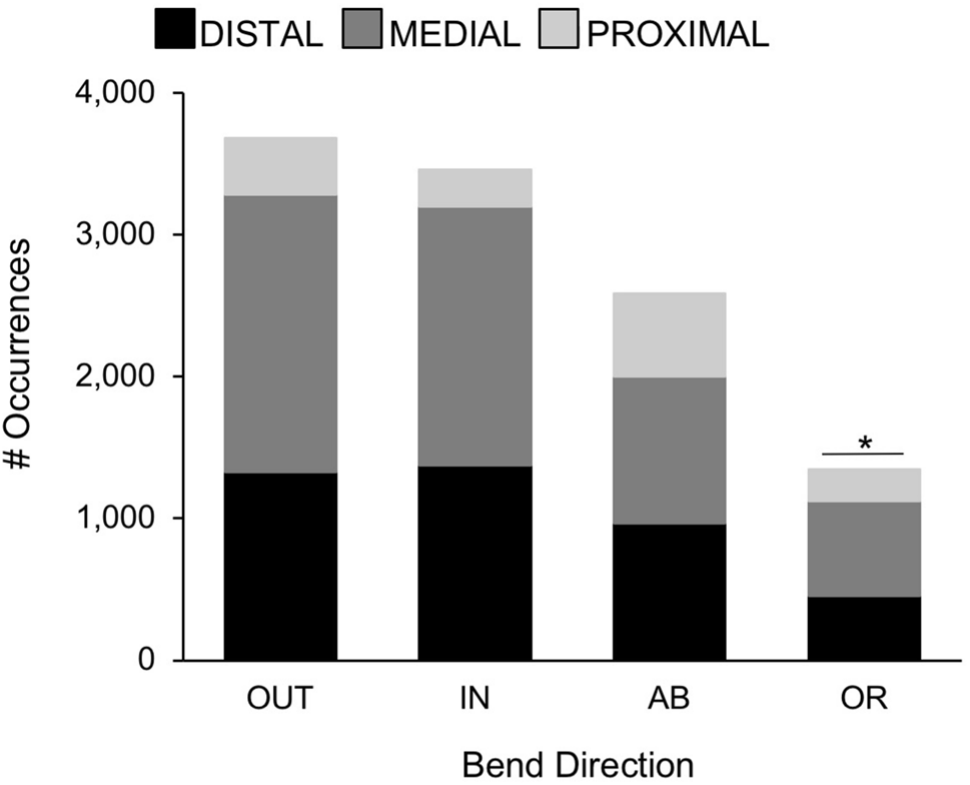
Bending comparisons in the arms of *O. bimaculoides*. Stacked bars representing the contributions of the distal, medial, and proximal arm regions are given for each of the four directions of bending. Oral bending was significantly lower than bending in other directions (X^2^ = 19.42, p = 0.001). Asterisks indicate significant differences in bending direction at p < 0.01.

**Figure 5:**
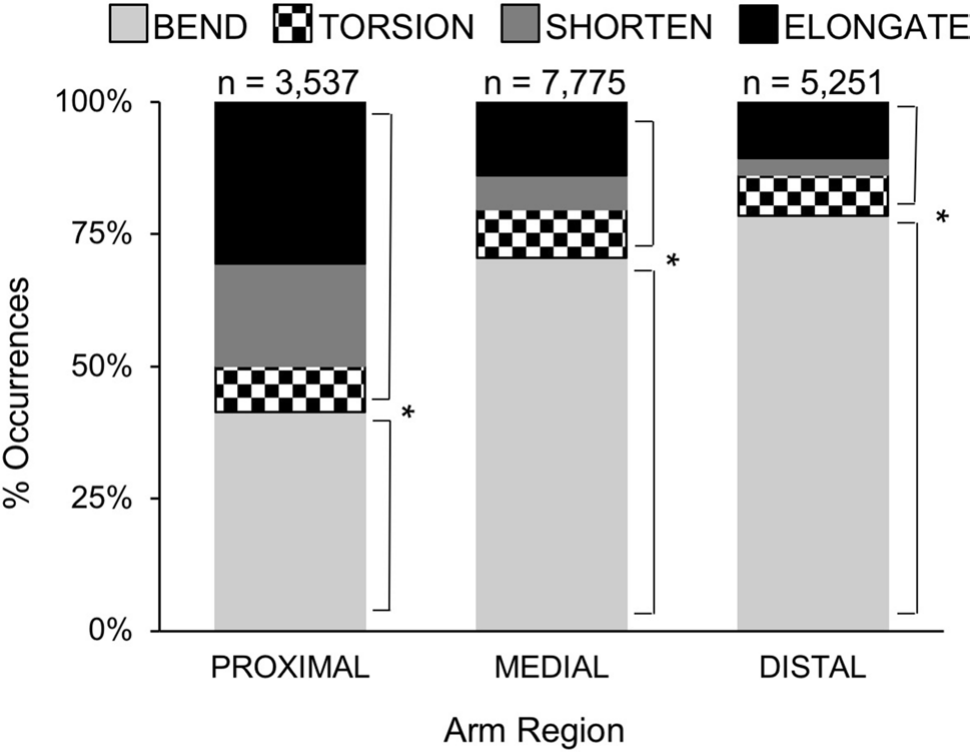
Percent distribution of deformation types among arm regions in *O. bimaculoides*. Bar graph showing the distributions of bending, torsion, shortening, and elongation within each arm region. Bending deformations made up the greatest proportion of the deformations recorded from the medial and distal arm regions (X^2^ = 4680.06, p = 0.001), while bending occurred less frequently in the proximal region as compared with other deformation types. Asterisks indicate significant differences in deformation type at p < 0.01.

Bending was the most common arm deformation of octopuses in our study: more than 11,000 examples were seen in the videos, the vast majority occurring in the anterior arms—i.e., arm pairs 1 and 2 (8,227 anterior arm bends, 2,847 posterior arm bends; Fig. 3A; X^2^ = 252.56, p = 0.006). Oral bending occurred less frequently than bending in any other direction (oral bends = 1,347 of 11,074 total bends; Fig. 4; X^2^ = 19.42, p = 0.001). Bending deformations made up the greatest proportion of the deformations recorded from the medial and distal arm regions (bends = 5,489 of 7,775 deformations; distal bends = 4,121 of 5,251 deformations; Fig. 5; X^2^ = 4680.06, p = 0.001) while bending occurred less frequently in the proximal region as compared with other deformation types (bends = 1,464 of 3,537 deformations; Fig. 5). Examples of local bending deformations can be viewed in Supplementary Video 1.

### Elongation and shortening

Elongation was observed 2,723 times and was seen more often in arm pairs 1 and 2 than pairs 3 and 4 (anterior = 1,887, posterior = 836; Fig. 3A; X^2^ = 112.40, p = 0.003). Shortening was observed 1,340 times; no significant differences were found in the anterior versus posterior arm pairs (anterior = 735, posterior = 605; Fig. 3A; X^2^ = 0.34, p = 0.55). Elongation and shortening deformations each constituted a greater proportion of the deformations recorded in the proximal base of the arms as compared to the distal and medial arm regions (Fig. 5; X^2^ = 4680.06, p = 0.002). Examples of elongation and shortening in the octopus arm can be viewed in Supplementary Video 1.

### Torsion

**Figure 6:**
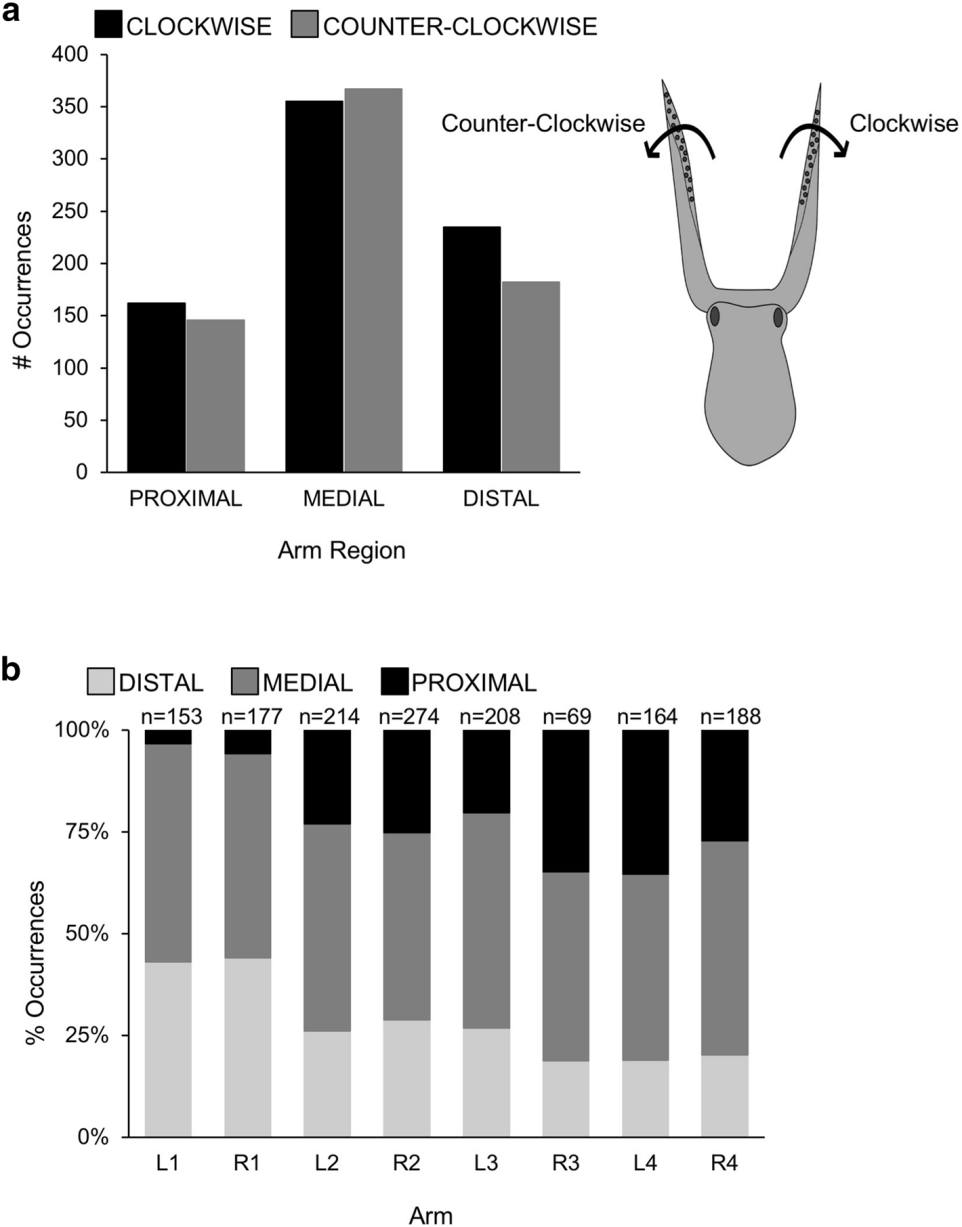
Torsion comparisons in the arms of *O. bimaculoides*. **(A)** The occurrences of clockwise and counterclockwise torsion are plotted with paired bars for each arm region (proximal, medial, and distal). A simple diagram illustrating the directions of torsion is included. No significant differences were found in the direction of torsion deformations in each arm region (X^2^ = 5.53, p = 0.06). **(B)** Relative percentages of occurrences of torsion are plotted in 100% stacked bars for each of eight arms. Torsion at the base of the arm was observed rarely in the first arm pair (15 occurrences of 1,426).

Torsion was observed 1,426 times and was not significantly different in directionality (clockwise torsion = 739, counterclockwise torsion = 687, Fig. 6A; X^2^ = 5.53, p = 0.06). Both clockwise (CW) and counterclockwise (CCW) torsion was observed in all three regions of the arms, with similar numbers of clockwise and counterclockwise torsion within the proximal region (CW = 161, CCW = 145, Fig. 6A; X^2^ = 5.53, p = 0.06), the medial region (CW = 346, CCW = 362, Fig. 6A), and the distal region (CW = 232, CCW = 180, Fig. 6A; X^2^ = 5.53, p = 0.06). There was no difference observed in the amount of torsion in anterior versus posterior arm pairs (X^2^ = 0.74, p = 0.39). Torsion in the proximal arm region was observed rarely in the first arm pair (15 occurrences of 1,426; Fig. 6B). Examples of clockwise and counterclockwise torsion are shown in Supplementary Videos 1, 2.

**Figure 7:**
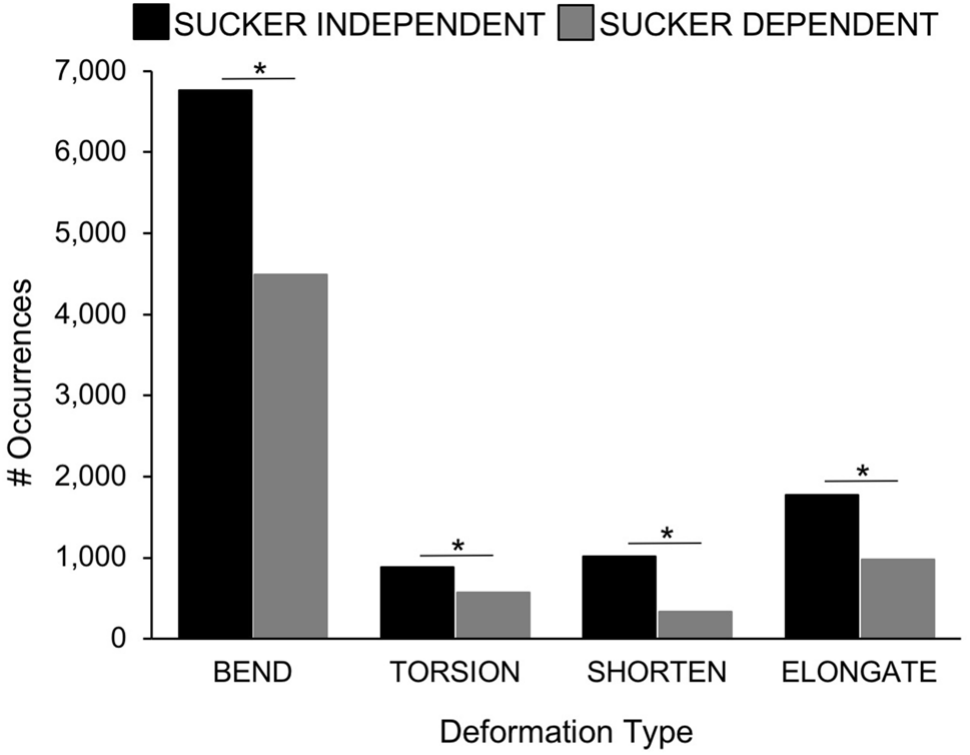
Sucker dependence for all deformations in *O. bimaculoides* arms. Occurrences of sucker-independent and sucker-dependent deformations are plotted in paired bars for each deformation type: bending, torsion, shortening, and elongation. All deformation types occurred more frequently without the use of suckers (X^2^ = 127.41, p = 0.008). Asterisks indicate significant differences in sucker use at p < 0.01.

All deformations occurred more frequently on the right than on the left side of the body (Fig. 3B; X^2^ = 10.49, p = 0.015), with the exception of the hectocotylized arm in males (R3 in Fig. 3B; p = 0.001). Additionally, all types of deformations occurred more often without the use of suckers, indicating that sucker use is not essential to the execution of any arm deformation (Fig. 7; X^2^ = 127.41, p = 0.008).

## Discussion

Our data support the long-standing assumption that octopus arms are one of the most flexible appendages found in nature. Field-caught *O. bimaculoides*, in response to a variety of situations requiring arm use in this laboratory setting, performed a wide range of broadly and highly localized arm movements using bending, torsional, elongating, and shortening deformations that totalled 16,563 separate events. The four deformation metrics we report here are essentially individual components of whole arm movements, and the videos provide a brief glimpse into the versatility and complexity of arm movements when such deformations are combined for varied behaviours (Supplementary Videos 1, 2). We discuss these findings with respect to the biomechanics of arm musculature, neural sensing and motor control, ecologically relevant behaviours, and bio-inspiration for soft robotics.

### Arm flexibility and behaviour

Octopus arms possess the needed intrinsic musculature to fully operate as muscular hydrostats, and we did observe this full range of capabilities in the arms of the octopuses in our study. All arms and arm regions were found to be capable of producing each of the observed deformations (Fig. 3), but some notable differences were observed in the relative execution of some deformations between arm regions. That is, (i) bending was the most common deformation observed (Fig. 3A), although orally directed bending was the least frequent (Fig. 4), (ii) most deformations were observed in the medial region of the arm (Fig. 5), and (iii) the proximal region of the arms tended to shorten and elongate more frequently relative to other deformation types (Fig. 5).

As a measure of flexibility, we began to look for portions of any arm that could not perform one of the basic deformations. All four deformation types (elongation, shortening, four directions of bending, and two directions of torsion) in each of three regions on each of eight arms amounts to a total of 192 possible local deformation combinations. Of these 192 possibilities, only one was never recorded; that is, proximal CCW torsion in arm R1. The remaining 191 possible deformations were observed at least once in all arms, suggesting that all combinations are possible hypothetically. However, proximal torsion in the first pair of arms was observed rarely in either direction (Fig. 6B). This could be due to the shorter front-most arms not needing frequent re-orienting or repositioning that can otherwise be accomplished with proximal torsion. In terms of flexibility ranges, we did not attempt to quantify the degree to which any deformation was performed, but we did observe impressive ranges of motion during video analysis (see Supplementary Videos 1, 2).

Although not representative of all behaviours, the distribution of movements among arms showed that the eight arms are not equally likely to be used for a given task (Fig. 3B). In general, anterior arms were used more frequently than posterior arms, and appeared to be the preferred arms used for exploring and manipulating objects presented. Byrne et al.^22^ found a similar arm preference in *O. vulgaris*, showing that anterior arms were used preferentially for exploring and collecting food; they also reported a lateral arm bias—octopus preferentially used a specific arm to retrieve food rewards in a T maze (however, some octopuses were found to prefer right arms while others preferred their left). A preference for the use of posterior arms for locomotion has also been suggested in some species^7,23,24^. Although the generalist hunting method of octopuses (using all eight arms and the interbranchial web to trap prey) suggests no particular ecological need for a task division among arms, a behavioural division of labour among arms may allow octopuses to hone specific skills.

Bending accounted for over 65% of all observations (11,074 of 16,563) and was the main deformation employed in creating arm movements. It is possible that bending may be the simplest method of moving a muscular hydrostat while keeping a proprioceptive understanding of body positioning. Octopus are thought to have relatively poor proprioceptive abilities and mainly use visual cues to approximate arm position^25^. Within the axial nerve cord of the octopus arm, proprioceptive activity is not transmitted longitudinally along the arm, although tactile activity does transmit this way^26^. With such local and distributed proprioception of the movements of eight individual arms, bending within a single plane may be more reliable without visual confirmation than other deformations. Based on extensive observation, we have concluded that one primary function of an octopus arm is to bring the suckers to bear on a surface. Seen throughout the video footage, reaching arms rotate so as to position the oral sucker surface of the arm to contact the target (Supplementary Videos 1, 2). Furthermore, bringing objects to the mouth (on the oral side of the octopus at the confluence of the eight arms) utilizes orally directed bending of the arm. However, in many of the filming situations in this study, animals did not have the opportunity to bring objects to their mouth because of dividers that only allowed arms through (Supplementary Video 1), and many animals did not choose to bring the inanimate objects to their mouths even when unrestricted. This may partially account for why oral bending was the least frequent bending direction observed in this study (Fig. 4). Additionally, within a given arm, most of the deformations were observed in the medial region (Fig. 5). If we consider a general function of the proximal region to be that of aiming the arm in a particular direction, and a general function of the distal region to be touching and sensing, then the medial region may deform more frequently by acting to connect the two, or by an overlap or coordination of functions between them. For example, one study found that the reaching movements of *O. vulgaris* were often directed to hit target surfaces with the central third of the arm^22^.

### Musculature

The anatomy of an octopus arm is composed almost entirely of muscle and connective tissue^27^ (Fig. 1), minimizing mechanical constraints of physical anatomy. However, there are some unique characteristics of the anatomy of octopus arms that raise questions about the muscular hydrostatic abilities of these limbs. Feinstein et al.^10^ showed that each arm muscle group in *O. vulgaris* differs in its density, orientation, and interaction with surrounding connective tissue, which itself generates different elastic forces passively. They noted that these differences described in the muscle anatomy may have an “important functional role in constraining the biomechanical properties of each muscle group” because the physiological properties have been found to be similar among them^17^. Moreover, the longitudinal muscle groups have collagenous tissue embedded in them and the functional significance of this is yet undetermined. These factors have also been suggested for cephalopod mantle and fin^28,29^ and may help to explain differences seen in our study in the relative execution of each of the deformations as these muscles work together in distinct activations to produce movement.

Aside from the intrinsic arm musculature that forms and operates the muscular hydrostat, octopus arms have additional anatomy and musculature that may affect the movement of the arm. For example, the acetabulo-brachial musculature that joins the suckers of the oral surface to the arm could inhibit structural flexibility and/or improve manoeuvrability through the use of suckers. Our observations suggest that suckers can increase the passive flexibility range of a given deformation (i.e., twist farther with the use of suckers; Supplementary Videos 1, 2), but sucker use is not critical to the execution of any arm deformation, as deformations occurred more often without the use of suckers (Fig. 7).

Stiffness, a deformation not measured in our study, has been shown to have a large effect on arm movement. Stiffening is commonly utilized by octopuses and is an important component of forming whole-arm movements^1^. For example, reaching movements are driven by a propagating wave of stiffening that propels an existing bend towards the distal end^14,30^.

### Neural control

Regardless of the physical capabilities of musculature, observable behaviours of freely moving octopuses are the result of neural and motor control of those muscles. The theoretical limitation of a system with “unlimited degrees of freedom” underlies the complexity of the neuromuscular control required. Understanding (and potentially replicating with robotics) such a system would require identifying mechanisms for simplifying control. Two discrete motor plans have been described in octopus (particularly *O. vulgaris*) arm movements thus far: reaching and fetching, and both were observed as described in *O. bimaculoides* during our analysis (Supplementary Video 2, 0:16). During reaching movements, a bend is propelled distally by a propagating wave of stiffening following an invariant velocity profile, in concert with elongation from the base of the arm^14,30–32^. Elongating the base of the arm functionally brings the rest of the arm and suckers closer to the intended target for sensing or manipulation, and this regional task division was reflected in our results (Fig. 5). The described motor plan for fetching—“pseudo joint fetching”—involves a quasi-articulated structure based on three dynamic joints in *O. vulgaris*^33,34^. Under constrained arm conditions (as in many of our lab conditions, Supplementary Video 1, 0:46), fetching is accomplished with a straight pull-in that may be a modified pseudo joint fetch, or a different motor program^35^. These motor plan descriptions were the results of complex video analyses and advanced mathematical and computer modelling, exceeding the abilities of our analysis. A finer-grained study could be accomplished by categorizing arm regions in finer fractions of sets of suckers, rather than by 1/3 arm segments as was done here to make data collection feasible. In the future, more refined methods, such as the complex methods developed by Yekutieli et al.^14^ and Zelman et al.^36^, could be used to collect more detailed experimental information describing full-scale deformations. Future experiments that use more precise video techniques combined with measurements of sucker position and orientation can expand our results and better inform biologists, biomechanists, neurobiologists and roboticists in future bio-engineering endeavors.

### Methodological considerations

The observational method used here was appropriate, effective, and practical for the study question^19,37,38^, yet it has limitations. The design was constructed to elicit movement from the octopus for the purposes of witnessing mechanical abilities rather than a representation of natural octopus behaviour. Our setup favoured frontal arms by design and octopus behaviour was affected by task-directed movement and other lab conditions related to our set up (e.g., the octopus was often separated from the object by a clear plexiglass divider). Our data collection on bending deformations was restricted to two orthogonal axes of movement—vertical and lateral—whereas naturally behaving octopuses are able to bend all points of their arms in all degrees in between, representing an unmeasured variation in our data. The filming methods did not allow for quantitative measurements of all eight arms simultaneously or the measurement of a fifth deformation, stiffening. However, we anticipated that any artefacts of our method would be largely outweighed by the sheer number of observations made here, and we hold that a more quantitative biomechanical approach would have hindered us in drawing any such general conclusions about arm flexibility.

### Bio-inspired robotics

The immense adaptability and complex fine control of octopus arms is of particular interest to materials scientists and engineers who deem the octopus arm to be an inspirational model for designing new soft robotic appendages^39–42^. As noted by Kier and Stella^1^, there is still a need for robots that can readily adapt to unstructured and cluttered environments with appendages that are multifunctional; for example, an arm that can be used for object identification and manipulation along its entire length. Many other applications in industry and medicine can be envisioned. Future research can also focus on the coordinated interplay of suckers and arm movements along each arm, and measurements of the degrees of freedom of flexibility in both of these organ types. The results of this study provide a starting point for studying what octopus arms are capable of and how these abilities could be translated into bio-inspired technology.

### Concluding thoughts

In the present study, our lab tank environment and filming set up were straightforward and relatively simple. Nevertheless, the curiosity of the octopuses produced an impressive variety of arm movements that can be appreciated by viewing our sample supplementary videos. Many avenues of inquiry remain for the future. Do the hatchlings of *O. bimaculoides* have the same full-arm capabilities as juveniles and adults? This species produces very large eggs and the hatchlings appear to be small adults in form and behaviour^43,44^; that is, there is no pelagic paralarval stage as in some other octopus species such as *O. vulgaris* (for which much is known about arm flexibility and control). It would be informative to obtain and analyse the range and diversity of arm flexibility from field studies of naturally behaving *O. bimaculoides* and other octopus species. Nevertheless, our data demonstrate that the octopus arm has exceptional flexibility. Although we did not see strong evidence that any region of *O. bimaculoides* eight arms could not perform all four observed deformations, it is possible that some limitations could be found if the octopuses were presented with more difficult manoeuvres; limitations and differences in arm movements may be found in other octopus species as well. In the laboratory, experimental manipulation challenges could be conceived to test details of arm movements, and more complex video arrangements that enable 3D interpretation of detailed arm flexibility would be welcomed. In the future, structured behavioural experiments in coordination with anatomical and neurophysiological studies will help unfurl the full potential as well as limitations of flexibility in octopus arms and may provide inspiration for future developments in soft robotics.

## Supplementary information

Supplementary Legends.

Supplementary Video 1.

Supplementary Video 2.

## Data Availability

Raw data will be made available at publication. The datasets are available from the corresponding authors on reasonable request.
