# Supplementary material for: Octopus arms exhibit exceptional flexibility
Source: Sci Rep. 2020 Nov 30;10:20872. doi: 10.1038/s41598-020-77873-7 (PMC7704652; doi:10.1038/s41598-020-77873-7)
Supplement: Supplementary file 1 — Supplementary Legends. [file 41598_2020_77873_MOESM1_ESM.docx]

**OCTOPUS ARMS EXHIBIT EXCEPTIONAL FLEXIBILITY.**

E.B. Lane Kennedy*^1^, Kendra C. Buresch^1^, Preethi Boinapally^2^, Roger T. Hanlon*^1^

^1^Marine Biological Laboratory, 7 MBL St, Woods Hole, MA 02543 USA

^2^Northeastern University, 360 Huntington Ave, Boston, MA 02115 USA

**SUPPLEMENTARY INFORMATION**

**Video 1. Examples of *Octopus bimaculoides* arm deformation types**. There are four video segments with a total duration of 1 minute 28 seconds.

**Video 2. Examples of complex arm movements in *Octopus bimaculoides***. There are six video segments with a total duration of 49 seconds.
